# Comparison of block and pulsed radiofrequency of the ganglion impar in coccygodynia

**DOI:** 10.3906/sag-1906-51

**Published:** 2019-10-24

**Authors:** Ender SİR, Sami EKSERT

**Affiliations:** 1 Department of Pain Medicine, Health Sciences University, Gülhane Training and Research Hospital, Ankara Turkey; 2 Department of Anesthesia and Reanimation, Health Sciences University, Gülhane Training and Research Hospital, Ankara Turkey

**Keywords:** Pulsed radiofrequency treatment, chronic pain, autonomic nerve block, coccygodynia

## Abstract

**Background/aim:**

Ganglion impar block is used for the treatment of chronic coccygodynia. Pulsed radiofrequency (PRF) of the ganglion impar is a promising novel technique. The aim of this study was to determine and compare the efficacy of the blockade and PRF of the ganglion impar.

**Materials and methods:**

Thirty-nine consecutive patients diagnosed with coccygodynia and treated with a blockade or PRF of the ganglion impar were included in this retrospective study. We compared the ganglion impar block (GIB) group (n = 25) with the ganglion impar pulsed radiofrequency (GIPRF) group (n = 14) in terms of pain intensity and patient satisfaction. We applied a numeric pain rating scale (NPRS) and a Likert scale (LS).

**Results:**

The NPRS scores in both groups had improved significantly from baseline at 3 weeks and at 3 and 6 months. However, in the sixth month, pain levels in the GIPRF group remained good, but they had returned to almost initial levels in the GIB group. Correspondingly, there were significant differences between groups in NPRS and patient satisfaction scores at 6 months (P ˂ 0.05).

**Conclusion:**

PRF neuromodulation provides significantly longer pain relief and reduces the risk of recurrence of pain in chronic coccygodynia as compared with blockade of the impar ganglion.

## 1. Introduction

Coccygodynia refers to pain and tenderness around the sacrococcygeal region. The most common etiologies are fracture, subluxation, and abnormal mobility of the coccyx. Patients usually have a previous history of trauma associated with coccygeal instability and this phenomenon triggers chronic inflammation around the sacrococcygeal joint [1,2].

The ganglion impar is a solitary retroperitoneal ganglion, which is formed by the termination of paired sympathetic chains in the sacral region [3]. It provides sympathetic and visceral innervation to the lower third of the rectum, vagina, vulva, urethra, anus, perineum, and coccyx [4]. The size and location of the ganglion on the anterior surface of the sacrococcygeal joint can vary. The ventral ramus of the sacral nerve roots can also run close to the ganglion [5]. Treatment of coccygodynia is challenging because of this anatomic variability and complex somatic, visceral, autonomic, and neuropathic components [6].

The most common symptom of coccygodynia is a pain in the sacrococcygeal region, which increases with sitting or standing up from a seat after a certain period. Although the presence of coccyx malposition on radiography may be useful in the diagnosis of coccygodynia, the main diagnosis is that the pain present in the coccyx region increases with palpation. The first step of treatment is conservative, which includes analgesic and antiinflammatory drugs, modifying the sitting style with the aid of ring-shaped pillows, manual manipulation of the coccyx, administration of transcutaneous electrical nerve stimulation, and physical therapy [7]. Each patient should be evaluated individually as no predictive factor has been identified for the most effective treatment modality [8]. In refractory patients, interventional treatment modalities, such as caudal epidural steroid injection, ganglion impar block (GIB), radiofrequency ablation, chemical neurolysis of the impar ganglion, and coccygectomy can be administered [9]. Blockade of the ganglion impar for the treatment of coccygodynia is an established procedure. However, the use of ganglion impar pulsed radiofrequency (GIPRF) for the treatment of coccygodynia is a relatively new approach, with limited results mostly from case reports and small series [10,11]. In this study, we aimed to compare the efficacy of GIB and GIPRF in the treatment of intractable coccygodynia by analyzing the long-term outcomes in these patients.

## 2. Materials and methods

This study was performed after obtaining approval from the institutional Gülhane Training and Research Hospital ethical committee (2019/01, 18/353). This study was designed retrospectively. We reviewed the medical records and follow-up forms of patients who were admitted to the Department of Pain Medicine between 1 August 2016 and 1 September 2018 with a diagnosis of chronic coccygodynia. The other inclusion criteria were intractable coccygeal pain for >3 months and unresponsiveness to conservative treatments, including nonsteroidal antiinflammatory drugs, topical local anesthetics, and physical therapy. The exclusion criteria were as follows: the presence of a local or systemic infection, history of allergic reactions to drugs and contrast dye, coagulopathy, use of an anococcygeal technique, patients with missing follow-up data, and previous history of coccygectomy.

A total of 39 consecutive patients treated with blockade or pulsed radiofrequency (PRF) of the ganglion impar were included in this study. All treatments were administered by the same physician who was experienced in GIB and GIPRF. All the patients provided written informed consent for the procedures. We assessed pain intensity using an 11-point numerical pain rating scale (NPRS): from 0, painless, to 10, worst imaginable pain [12]. We evaluated patient satisfaction with a Likert scale (LS), which allows the patients to define the level of agreement or disagreement (≥3, satisfied; ≤2, unsatisfied) [13] at 6 months after the procedures. We assessed NPRS scores at the time of presentation and at 3 weeks, 3 months, and 6 months after the block, as well as patient satisfaction at 6 months after the block. We collected all data from the patients’ medical records and follow-up forms.

### 2.1. Technique

The patients were placed in prone positions with a cushion beneath the abdomen to properly view the sacrococcygeal disc. We performed routine noninvasive monitoring of blood pressure, pulse oximetry, and electrocardiography, and established intravenous access. After aseptic preparation using 10% povidone-iodine, we infiltrated the skin with 2% lidocaine. 

**Figure 1:**
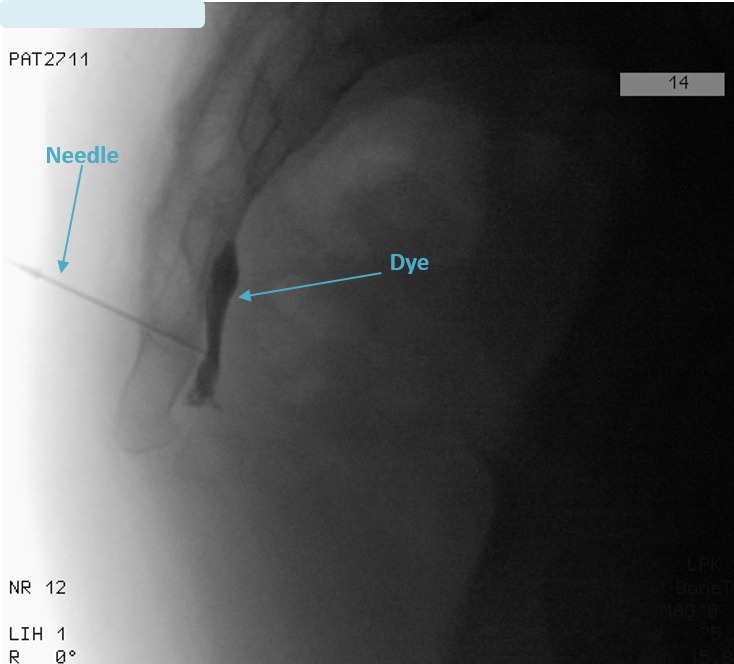
Needle position for the ganglion impar block/PRF: lateral view with contrast dye indicating the location of the impar ganglion.

In the GIB group, we introduced a 22-G spinal needle through the sacrococcygeal disc under fluoroscopic guidance along with anteroposterior and lateral imaging. We then administered radiopaque dye to confirm the accurate location of the needle tip when it reached the anterior surface of the sacrococcygeal region in the retroperitoneal space (Figure 1). After negative aspiration of blood and cerebrospinal fluid, we injected 2 mL of 0.25% bupivacaine with 1 mL (40 mg) of triamcinolone acetate, and then withdrew the needle slowly. 

In the GIPRF group, we introduced a 22-G, 10-mm active-tip radiofrequency cannula through the sacrococcygeal disc under fluoroscopic guidance. When the needle tip reached the anterior surface of the sacrococcygeal disc, we injected a radiopaque dye to confirm the needle’s position. Before performing PRF, we checked the tissue impedance, motor, and sensory responses. The expected tissue impedance was <500 ohm. The feeling of sensorial paresthesia around the sacrococcygeal region was <1 V at 50 Hz. We performed PRF at 42 °C for 120 s for 3 cycles with standard clinical PRF parameters (voltage, 45 V; pulse rate, 2 Hz/s; pulse width, 1 ms). No medication was injected during the GIPRF procedure. All adverse events and complications were recorded on the follow-up forms.

### 2.2. Statistical analyses

All analyses were performed with SPSS 21.0. Descriptive statistics (number, percentage, mean, standard deviation, minimum, and maximum values) were used. Wilcoxon’s test was used to compare the continuous data in the dependent binary groups that were not normally distributed. The Mann–Whitney U test was used to compare the continuous data in independent binary groups, and Fischer’s final test was used to compare the discrete data. P < 0.05 indicated statistical significance.

## 3. Results

We reviewed a total of 44 files of patients who were admitted to our clinic with chronic coccygodynia. The trans-sacrococcygeal approach was undertaken for all the patients. We excluded 2 patients in whom the anococcygeal approach was undertaken, 2 patients who had a prior history of coccygectomy, and 1 patient with missing follow-up data. We administered GIB to 25 patients and GIPRF to 14 patients. The sociodemographic characteristics were similar in both the groups: 72% of the patients were female, the mean age was 44.6 years, and 77% of the patients had an antecedent trauma history (Table 1). 

**Table 1:** Demographic profile of the patients.

	Group GIB (n: 25)	Group GIPRF (n: 14)	P
Age (mean ± SD), year	42.64 ± 13.60	45.52 ± 14.68	0.43
Weight (mean ± SD), kg	72.45 ± 13.96	75.63 ± 15.23	0.59
BMI (mean ± SD)	24.73 ± 4.56	27.98 ± 6.83	0.52
Fracture-malposition (%)	19 (76)	11 (78.5)	0.75

GIB: Ganglion impar block, GIPRF: ganglion impar pulsed radiofrequency, BMI: body mass index, SD: standard deviation.

**Figure 2:**
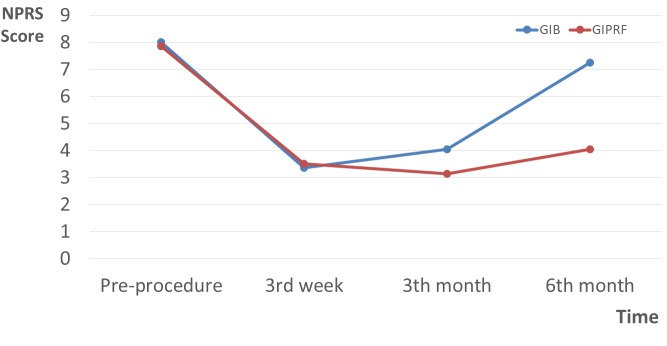
NPRS scores in the groups (GIB: ganglion impar block, GIPRF: ganglion impar pulsed radiofrequency).

In the GIB and GIPRF groups, the mean NPRS scores were 8.00 to 7.85 before injection, 3.36 to 3.50 in the third week, 4.04 to 3.14 in the third month, and 7.24 to 4.05 in the sixth month (Figure 2). There was a significant difference between groups in terms of NPRS scores at 6 months (P = 0.03). The LS scores for patient satisfaction at the sixth month in the GIB and GIPRF groups were 48% and 71.4%, respectively (P ˂ 0.001) (Table 2). We detected hypotension and bradycardia in one patient of the GIB group, which was treated with 0.5 mg of atropine intravenously in the follow-up period. No other complication or side effect was observed in either group.

**Table 2:** Numeric pain rating scale (NPRS) and Likert scale (LS) scores of the patients before and after the treatment.

	Group GIB (n: 25)	Group GIPRF (n: 14)	P*	P**	P***
Baseline NPRS (mean ± SD)	8.00 ± 1.29	7.85 ± 1.61	-	-	0.77
3rd week NPRS (mean ± SD)	3.36 ± 3.08	3.50 ± 3.39	˂0.001	0.005	0.98
3rd month NPRS (mean ± SD)	4.04 ± 3.22	3.14 ± 3.52	˂0.001	0.005	0.34
6th month NPRS (mean ± SD)	7.24 ± 2.92	4.05 ± 3.89	0.41	0.02	0.03
6th month LS (%)	12 (48)	10 (71.4)	-	-	˂0.001

GIB: Ganglion impar block, GIPRF: ganglion impar pulsed radiofrequency, SD: standard deviation.

* Significance of the GIB group from preprocedural values.

** Significance of the GIPRF group from preprocedural values.

*** Significance between the GIB group and the GIPRF group.

## 4. Discussion

Blockade of the ganglion impar is an effective pain-relieving procedure used for many years for the management of coccygodynia [14]. However, PRF of the ganglion impar is a relatively novel approach for this indication [6]. In the present study, we achieved excellent pain relief in both GIB- and GIPRF-administered patients in the first three months. Nevertheless, while the pain in the GIB group started to return to baseline pain levels, the GIPRF group continued to maintain improved pain levels. 

PRF is a nondestructive approach that has been used for >20 years in the field of pain management, and there is increasing interest in its use recently [15–17]. Anatomically, if we begin from the head region, we can first give examples of its use in occipital neuralgia and cervicogenic headaches [18]. Subsequent treatments include suprascapular, intercostal, dorsal root ganglia, and other peripheral nerves [19,20]. Apart from its use in nerves, there are also studies about the intraarticular and intralesional use of PRF [21,22]. The main advantage of PRF is that it provides long-term pain control without complications [23]. Hence, in our study, we used GIPRF as an alternative to GIB to provide long-term pain relief with a low risk of complications. The underlying mechanisms of neuromodulator effects of PRF are still not fully clarified. It creates an electrical field around the active tip of the needle, which penetrates into the interior of the axons. Although the target tissue temperature is set at 42 °C, which is below the tissue destruction threshold of 45 °C to 50 °C [24], ultrastructural changes in the nociceptive axons are seen, especially in the pain-carrying A-delta and C-fibers [25]. Hagiwara et al. suggested that the analgesic effects of PRF are associated with enhancement of noradrenergic and serotonergic descending pathways [26]. Moreover, Van Zundert et al. identified an increased number of c-Fos-immunoreactive cells in the dorsal horn. Alterations of these gene products trigger long-term changes in gene expression, which is responsible for the long-term potentiation [27]. In accordance with our results, these changes might explain the long-lasting antinociception after PRF neuromodulation in the GIPRF group.

For blockade and PRF of the ganglion impar, transdiscal sacrococcygeal, paramedian sacrococcygeal, and anococcygeal approaches can be attempted [28]. We used the transdiscal sacrococcygeal technique because of its low complication risk and ease of administration under fluoroscopic guidance [3]. It is almost impossible to reach the ganglion in cases where the sacrococcygeal discs are calcified and sacrococcygeal joints are fused. In the current study, we inducted a sacrococcygeal approach, and we excluded two patients in whom we could not reach the ganglion and used anococcygeal approach for them.

There are variations in the anatomical locations and shapes of the ganglion impar. Furthermore, the ventral ramus of the sacral nerves can run close to the ganglion. To prevent complications such as nerve damage or neuritis, we administered GIB with local anesthesia and steroids or GIPRF instead of using radiofrequency ablation or chemical neurolytic agents [5]. According to our study, we revealed an increasing trend of improvement in long-term pain scores in the GIPRF group. Further randomized controlled studies evaluating the efficacy of GIPRF and GIB should be performed to establish an evidence-based gold-standard treatment for coccygodynia. The main limitation of this study was its retrospective design. We evaluated improvement in pain relief and patient satisfaction. Improvement in functional disabilities could be evaluated, as well. Nevertheless, our study makes a significant contribution to the literature because, to the best of our knowledge, this is the first study to compare the efficacy of block and PRF of the ganglion impar for the treatment of coccygodynia.

To conclude, both the block and the PRF of the impar ganglion in coccygodynia improve pain in short-term and mid-term periods. However, in light of our preliminary study, the long-lasting effect of neuromodulation with GIPRF provides better pain relief than GIB in the long term.
